# Spinal Accessory Nerve Schwannoma in a Pediatric Patient: A Rare Microcystic/Reticular Variant in a Non-syndromic Case

**DOI:** 10.7759/cureus.105518

**Published:** 2026-03-19

**Authors:** Aldair O De Los Reyes-Lara, Jonathan Ortiz-Rafael, Ricardo Gonzalez-Fernandez, Jorge A Garcia Garza, Jesus G Lozano Cadena, Isaac J Palacios-Ortiz

**Affiliations:** 1 Department of Neurological Surgery, Hospital Regional de Monterrey Instituto de Seguridad y Servicios Sociales de los Trabajadores del Estado (ISSSTE), Monterrey, MEX; 2 Department of Neurological Surgery, Hospital Universitario Dr. José Eleuterio González Universidad Autónoma de Nuevo León (UANL), Monterrey, MEX; 3 Department of General Surgery, Hospital Regional de Monterrey Instituto de Seguridad y Servicios Sociales de los Trabajadores del Estado (ISSSTE), Monterrey, MEX

**Keywords:** far-lateral approach, microcystic schwannoma, schwannoma surgical management, spinal accessory nerve, spinal accessory nerve schwannoma

## Abstract

Neoplasms of the craniocervical junction in the pediatric population are rare pathologies that represent a therapeutic challenge. We present the case of a 10-year-old male patient with a two-month history of upper limb weakness and gait disturbance. Magnetic resonance imaging revealed a lesion in the foramen magnum. Microsurgical resection was performed via a far-lateral approach, identifying the origin in the spinal accessory nerve. Although initial histopathology suggested a World Health Organization grade 2 chordoid meningioma, immunohistochemistry confirmed a microcystic/reticular-variant schwannoma. Following complementary treatment with radiotherapy due to the initial histological suspicion of a grade 2 meningioma and the complex ventral location of the lesion, the patient showed complete neurological recovery and absence of residual lesion at nine months, highlighting the importance of immunohistochemical diagnosis in determining therapeutic management and prognosis.

## Introduction

Craniocervical junction neoplasms in the pediatric population constitute a heterogeneous group of rare pathologies whose therapeutic approach represents a significant challenge due to their complex relationship with critical neurovascular structures. Within this group, schwannomas originating from lower cranial nerves or upper spinal nerves are sporadically reported in the literature, especially in patients without a syndromic history [1], as an association with neurofibromatosis has been established.

Intracranial schwannomas comprise 8% of primary intracranial tumors, and more than 90% of intracranial schwannomas originate from the vestibular nerves. Those originating from non-vestibular nerves are rare. Accessory nerve schwannomas constitute 6.66% of all non-vestibular schwannomas [2], mostly occurring in the adult population with extracranial or spinal localization.

From a clinical perspective, these tumors can manifest with non-specific symptoms, which delay diagnosis. General symptomatology, such as headache and cervicalgia, occurs in 70% of cases; signs of cervical radiculopathy are present in 40%, and progressive paresthesia of the extremities is found in 22% [3]. Differential diagnoses should include other foramen magnum neoplasms, such as meningiomas or chordomas [1].

Spinal accessory nerve (SAN) schwannomas are exceedingly rare clinical entities. According to the systematic review by Yan and Wang, only 62 cases had been documented in the global literature as of 2020 [4]. These tumors are even less frequent in the pediatric population and represent a significant diagnostic challenge due to their nonspecific clinical presentation and the complex anatomy of the craniocervical junction, often mimicking other pathologies such as chordoid meningiomas [4,5].

The objective of the present study is to report an unusual case of a SAN schwannoma in a non-syndromic 10-year-old pediatric patient, highlighting the significance of the microcystic/reticular histological variant. We emphasize the diagnostic transition from the initial suspicion of a meningioma to the definitive confirmation through immunohistochemical analysis (S-100 protein), while also discussing the far-lateral approach to achieve complete resection in the pediatric age group. This specific histological subtype is characterized by prominent cystic spaces and a lace-like reticular growth pattern within a myxoid stroma. Due to these features, the microcystic/reticular variant can be easily misinterpreted as other myxoid neoplasms or higher-grade tumors, such as chordoid meningiomas or chordoid gliomas, making immunohistochemical differentiation essential for an accurate diagnosis [5].

## Case presentation

A 10-year-old male patient with no chronic history presented for evaluation due to upper limb weakness and gait disturbance of two months’ evolution, which prevented him from performing normal sports activities. Upon admission, physical examination revealed he was alert with a score of 15 on the Glasgow Coma Scale [6] and with 4/5 motor strength in both upper limbs according to the Daniels and Worthingham’s scale for muscle testing [7]. A meticulous cranial nerve assessment was performed. Despite the tumor’s location, the gag reflex was preserved, and tongue protrusion was symmetric, indicating no involvement of the IX, X, or XII nerves. No facial asymmetry or hearing deficits were noted. Furthermore, motor strength in the lower extremities was 5/5, and sensory functions remained intact. Deep tendon reflexes were normal across all four limbs, with no signs of hyperreflexia, Hoffman’s, or Babinski’s signs, as is often observed in localized accessory nerve lesions [8].

**Figure 1:**
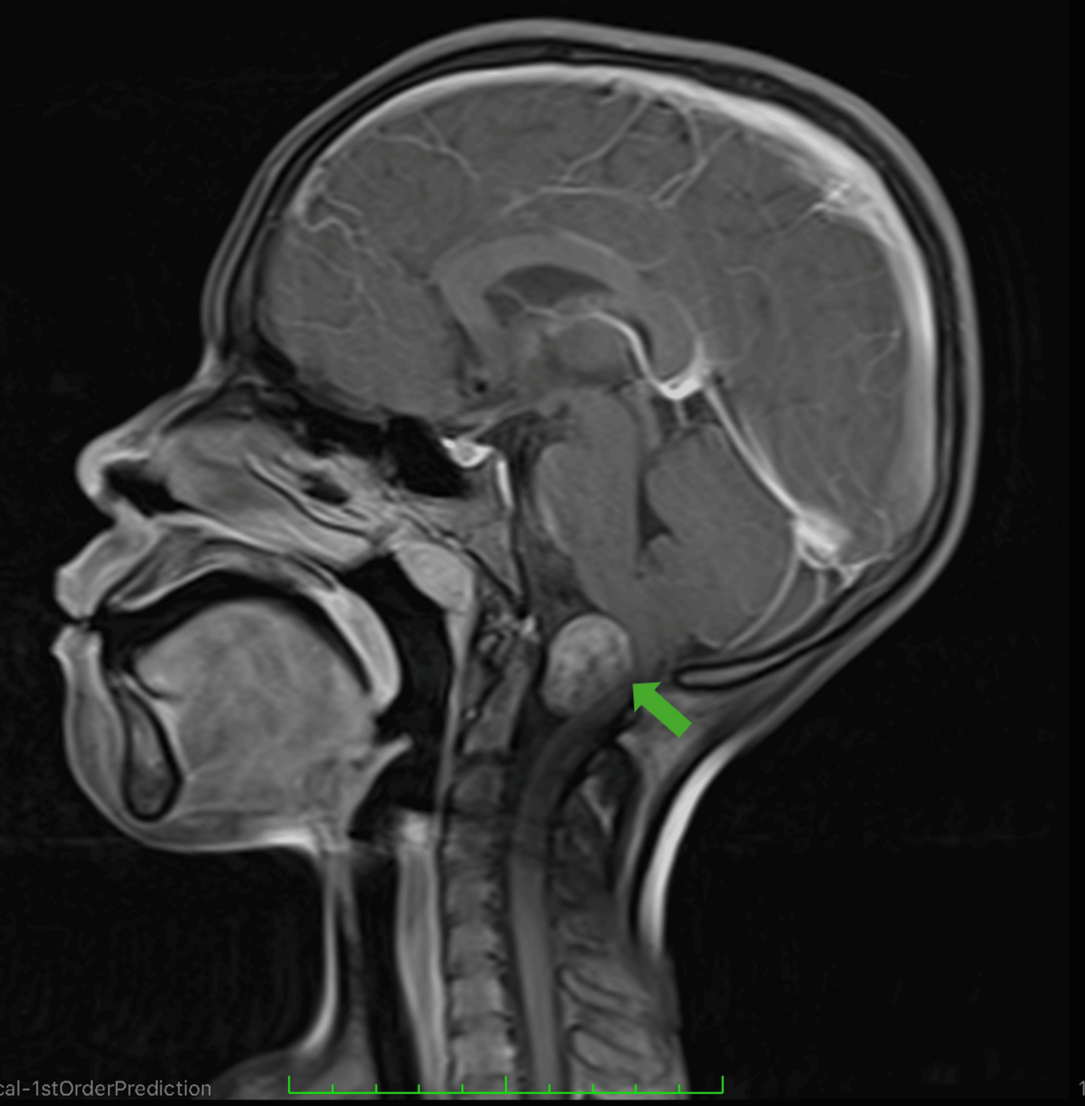
Preoperative neuroimaging. Contrast-enhanced magnetic resonance imaging (gadolinium) of the brain and craniocervical junction showed the lesion was hyperintense on T2 and had an oval-shaped image with avid and heterogeneous enhancement, measuring 2.5 × 2 × 2 cm in its longitudinal, anteroposterior, and transverse diameters (green arrow), and was displacing the medulla oblongata posteriorly.

Magnetic resonance imaging (MRI) of the brain and craniocervical junction was performed. The study identified a well-circumscribed, oval-shaped intradural extramedullary lesion measuring 2.5 × 2 × 2 cm. The mass exhibited an isointense signal on T1 sequences and was hyperintense on T2 images. Following gadolinium administration, the lesion showed avid and heterogeneous enhancement. The tumor was located ventrally at the level of the foramen magnum, causing significant posterior displacement and compression of the medulla oblongata and the upper cervical spinal cord. No evidence of syrinx formation or obstructive hydrocephalus was identified (Figure 1).

**Figure 2:**
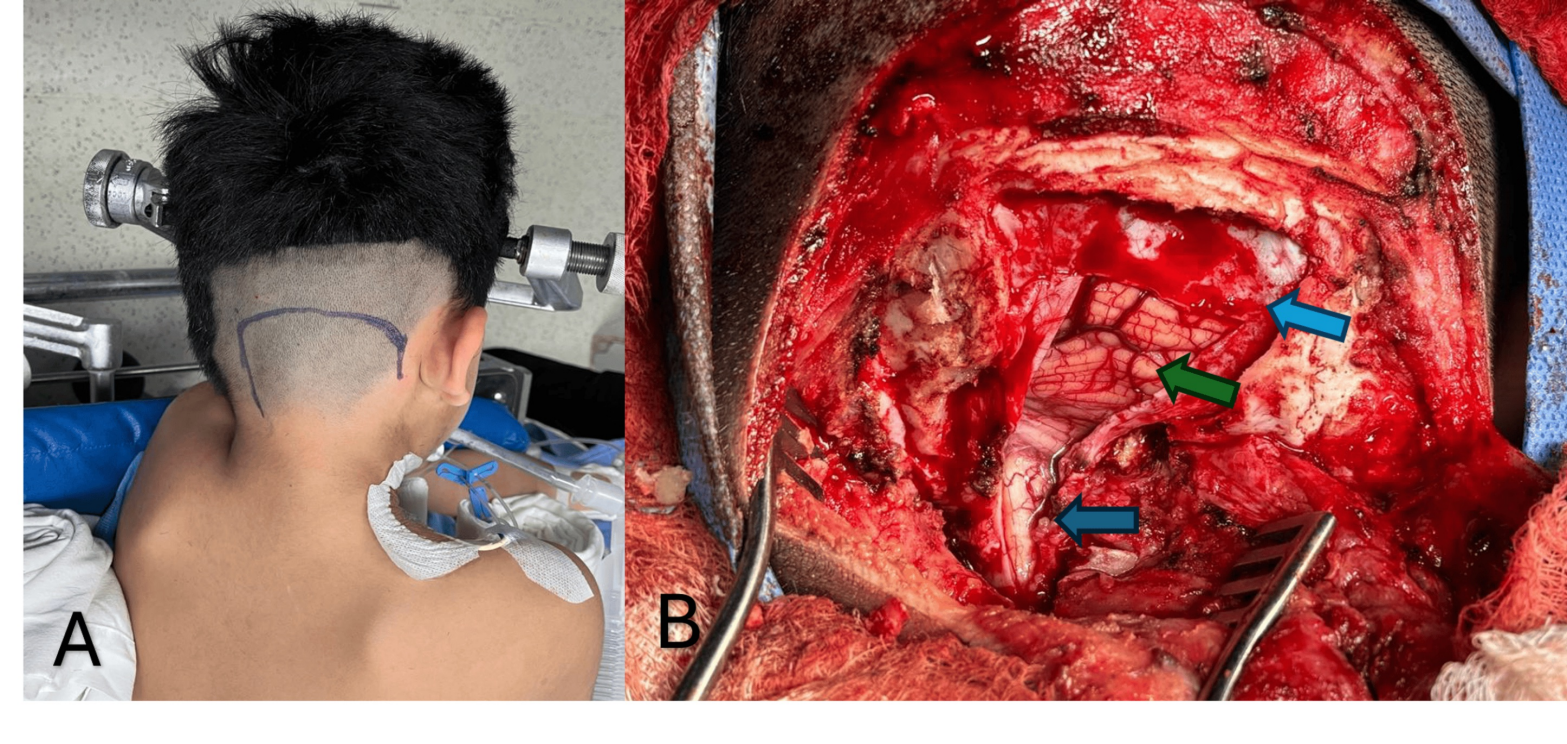
Surgical approach. (A) Far-lateral approach with a horseshoe-type incision. (B) Exposure following craniotomy and resection of the C1 posterior arch. Blue arrow: brainstem; green arrow: cerebellum; light blue arrow: dura mater.

**Figure 3:**
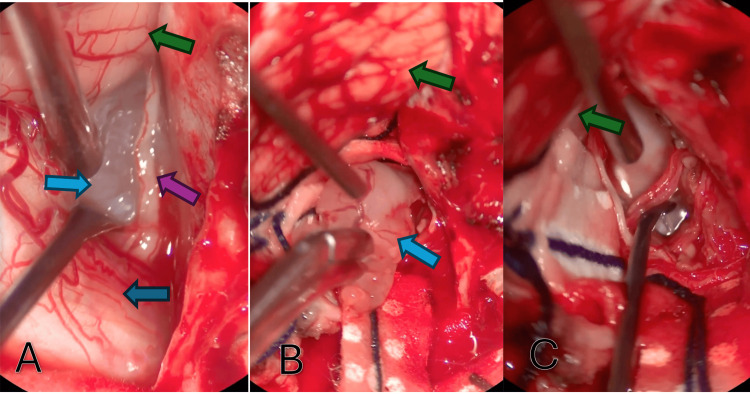
Intraoperative findings. (A) Initial approach exposing the tumor by moving the medulla oblongata; the cerebellum is shown on the upper left side and the C1 accessory branch is seen on the right side of the tumor. (B) Tumor extraction and cerebellum. (C) Final surgery result with extraction of the tumor. Light blue arrow: tumor; blue arrow: medulla oblongata; green arrow: cerebellum; purple arrow: C1 accessory branch. Images were captured using a ARveo 8× 3D Digital Microscope.

A surgical approach was decided upon for resection and diagnosis. A far-lateral approach was performed in a left-sided park-bench position using a horseshoe incision (Figures 2A, 2B). A whitish, rubbery, foramen magnum lesion was observed (Figures 3A, 3B), which was difficult to aspirate and showed a portion firmly adhered to the spinal accessory nerve (XI). Complete resection was achieved, except for the portion adhered to the nerve (Figure 3C).

**Figure 4:**
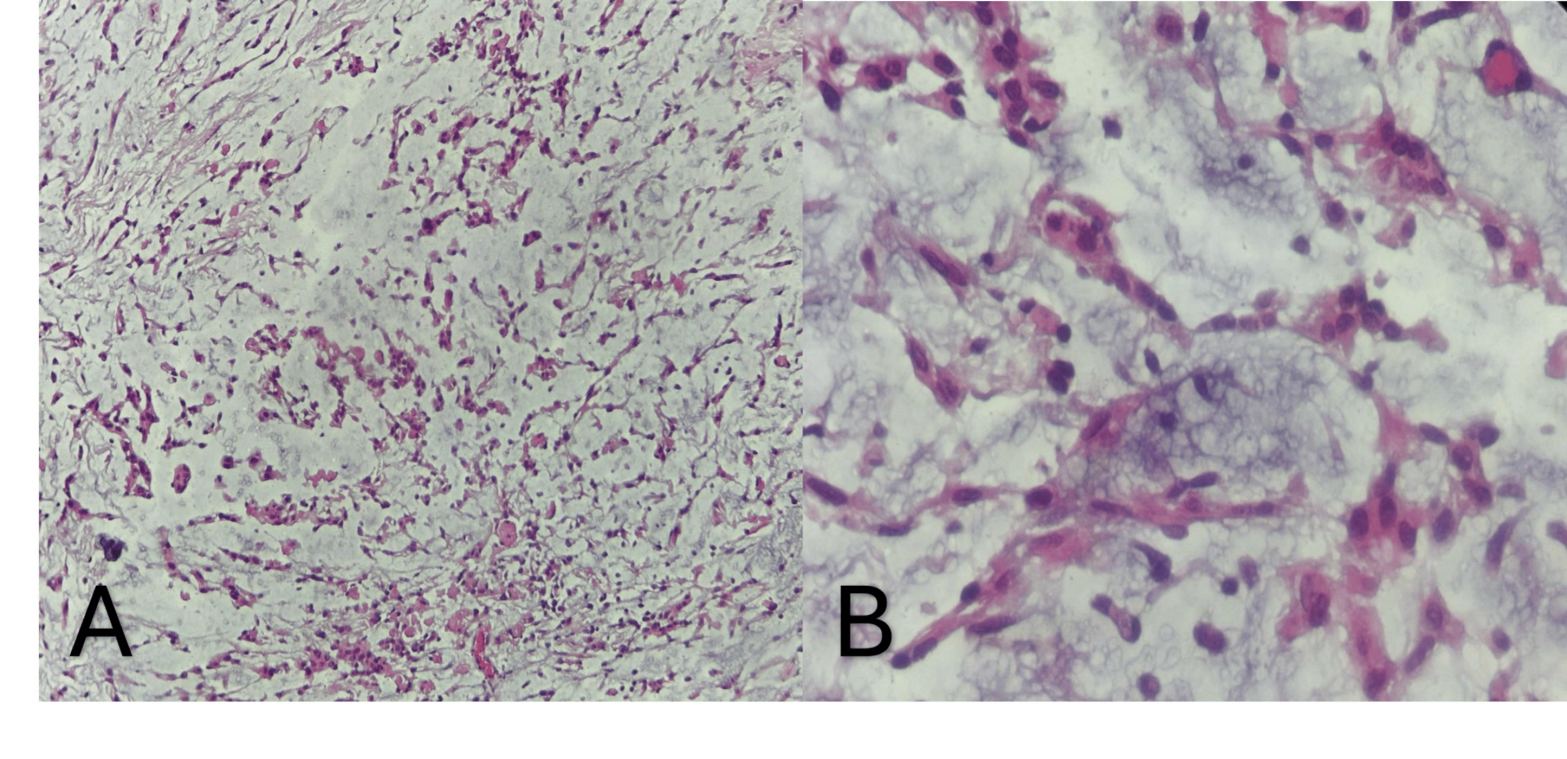
Histopathological analysis. (A) Low-magnification view showing groups of round cells with small nuclei and eosinophilic cytoplasm, predominantly arranged in cords and rows. (B) High-magnification microscopic view highlighting the tumor cells forming nests and reticular patterns within a prominent pale myxoid-appearing matrix.

The patient remained hospitalized for six days post-surgery before being discharged. At the 15-day follow-up, he presented with 5/5 strength on the Daniels scale and gait improvement. The histopathology study reported a World Health Organization (WHO) grade 2 chordoid meningioma (Figure 4). Complementary immunohistochemistry revealed diffuse S100 positivity and focal glial fibrillary acidic protein (GFAP) positivity, compatible with a microcystic/reticular-variant schwannoma.

**Figure 5:**
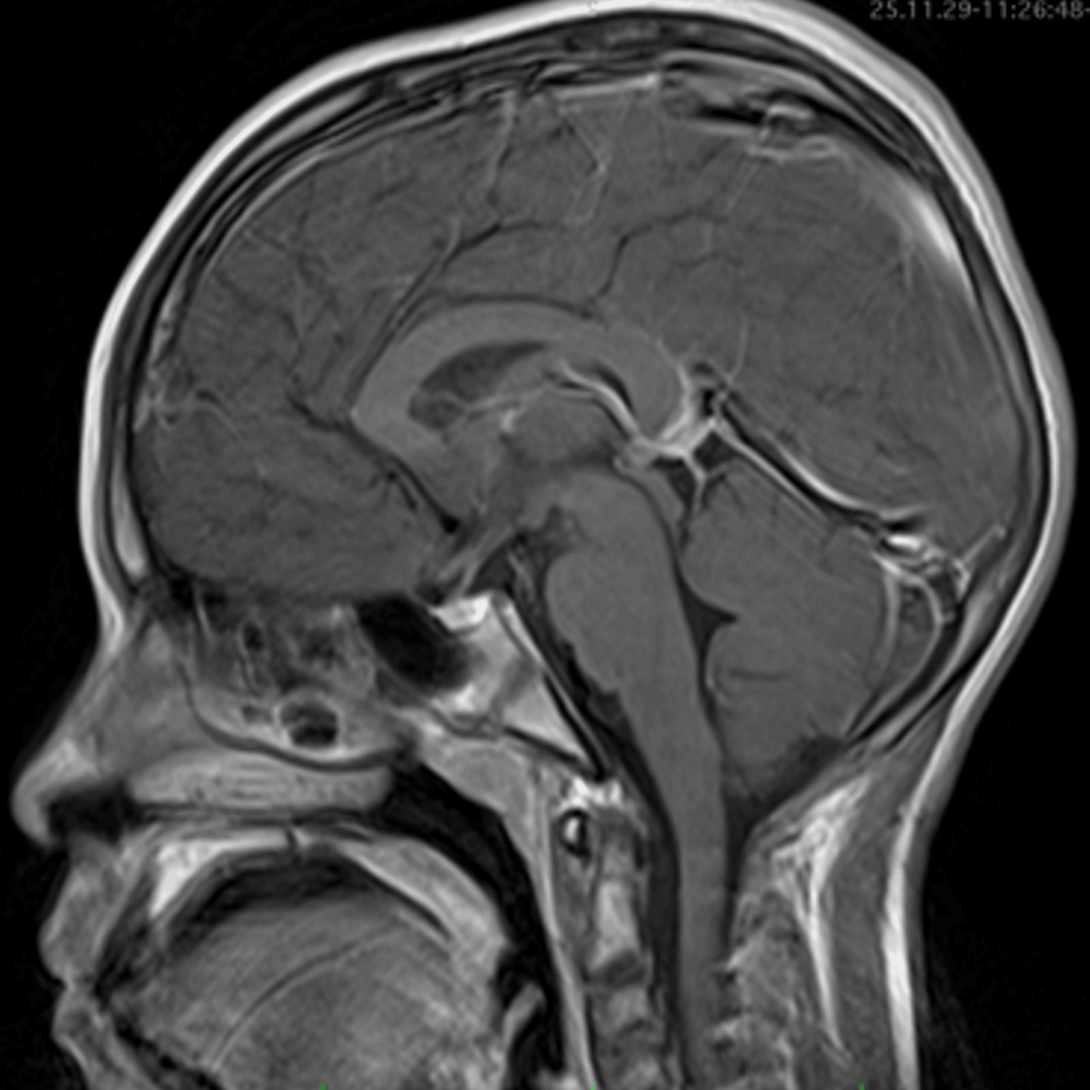
Postoperative follow-up. Postoperative magnetic resonance imaging showing complete surgical resection.

Subsequently, radiotherapy was administered with a planning target volume 1 dose of 45.0 Gy in 25 sessions. The patient returned for follow-up with a contrast-enhanced MRI at nine months post-surgery (Figure 5), showing no evidence of residual lesion. He was referred to medical genetics to rule out associated syndromes, which were discarded.

## Discussion

Schwannomas are benign tumors derived from Schwann cells that are rare in the pediatric age group. Overall, 90% originate from the vestibular nerve; among non-vestibular cases, only 6.66% involve the accessory nerve (XI) [2]. Most cases described in the literature correspond to adult patients, with a slight male predominance [4]. In the pediatric population, besides their low frequency, there is a relevant association with genetic syndromes such as neurofibromatosis type II [1]. In contrast, the present case involved a pediatric patient without clinical or genetic data of syndromic disease, underlining the rarity of this presentation.

In foramen magnum tumors, headache or cervicalgia has been reported in up to 70% of cases, myelopathy in approximately 50%, and lower cranial nerve involvement in around 38% [3]. In clinical series of foramen magnum lesions, pain, paresthesias, and myelopathy remain the hallmark symptoms due to spinal cord compression [4].

Anatomically, while most lesions are dorsal, ventral schwannomas, as in the present case, represent a significant surgical challenge due to their proximity to the brainstem [3]. Ventral lesions, as in the present case, are less frequent and represent a greater surgical challenge due to proximity to the brainstem and neurovascular structures. The far-lateral approach is currently the preferred surgical strategy for these ventral craniocervical junction lesions, as it provides optimal exposure and neurovascular control [1,3]. In our case, this approach enabled macroscopically complete resection with functional preservation of the accessory nerve.

Preoperative identification of the nerve of origin through imaging is limited. In most cases, the definitive diagnosis of the involved nerve is established during the surgical act [2,8]. In this patient, the firm tumor’s adherence to the spinal accessory nerve allowed intraoperative confirmation of its origin.

The use of adjuvant radiotherapy in benign schwannomas remains controversial; however, in this case, the decision was multifaceted. First, the initial pathological diagnosis suggested a WHO grade 2 chordoid meningioma, a lesion known for higher recurrence rates that typically warrants adjuvant radiation. Second, the ventral location of the tumor at the craniocervical junction poses a significant surgical challenge, where adjuvant therapy is often utilized to arrest the growth of any microscopic or subtotal residual tissue near the brainstem, as supported by current management paradigms for complex skull base lesions [1,8].

Regarding histopathology, schwannomas are well-circumscribed tumors composed of a clonal population of Schwann cells, characterized by diffuse nuclear and cytoplasmic immunoreactivity for the S100 protein [5]. Initially, the histopathological study suggested a WHO grade II chordoid meningioma, a diagnosis implying a distinct and potentially more aggressive biological behavior. However, in the present case, immunohistochemistry evidenced S100 and GFAP positivity, compatible with the microcystic/reticular variant, a morphological presentation reported as unusual even in series dedicated to pediatric non-vestibular schwannomas [9]. This situation highlights the importance of immunohistochemical analysis in cases with morphological overlap, especially when the initial diagnosis could substantially modify therapeutic conduct and prognosis.

Complete microsurgical resection is the treatment of choice for accessory nerve schwannomas [2,4]. In extracranial XI nerve schwannomas, nerve sacrifice has been reported in up to 56% of cases, with risk of permanent or transient deficits [8]. In our case, it was possible to preserve the nerve, achieving complete neurological recovery in early follow-up and absence of radiological recurrence at nine months.

## Conclusions

Accessory nerve schwannoma in the pediatric population is an extremely unusual entity, especially in the absence of neurofibromatosis. This case underscores that when facing foramen magnum tumors with non-specific clinical presentations, such as motor weakness or gait disturbance, it is vital to consider this differential diagnosis alongside more common neoplasms. The successful management of this patient demonstrates that while the far-lateral approach provides an effective surgical corridor for ventral lesions at the craniocervical junction, the clinical outcome is heavily dependent on a multidisciplinary strategy. Furthermore, the critical role of immunohistochemistry is emphasized; S100 protein positivity was decisive in rectifying the initial diagnosis of chordoid meningioma. This differentiation is essential when dealing with rare histological subtypes, such as the microcystic/reticular variant, which can mimic more aggressive tumors due to their myxoid matrix. Correct identification avoids an erroneous prognosis and ensures appropriate therapeutic management. The patient’s favorable evolution confirms that meticulous microsurgery, supported by precise histopathological and immunohistochemical analysis, is fundamental for achieving total neurological recovery.
